# Supplementary material for: Inhibition of human-HPV hybrid ecDNA enhancers reduces oncogene expression and tumor growth in oropharyngeal cancer
Source: Nat Commun. 2025 Mar 26;16:2964. doi: 10.1038/s41467-025-57447-9 (PMC11947173; doi:10.1038/s41467-025-57447-9)
Supplement: Supplementary file 1 — Supplementary Information [file 41467_2025_57447_MOESM1_ESM.pdf]

Inhibition of human-HPV hybrid ecDNA enhancers reduces oncogene expression and tumor growth in oropharyngeal cancer

Takuya Nakagawa, Jens Luebeck, Kaiyuan Zhu, Joshua T. Lange, Roman Sasik, Chad Phillips, Sayed Sadat, Sara Javadzadeh, Qian Yang, Abdula Monther, Santiago Fassardi, Allen Wang, Kersi Pestonjamasp, Brin Rosenthal, Kathleen M. Fisch, Paul Mischel, Vineet Bafna, & Joseph A. Califano

Supplementary Figures:

Supplementary Fig. 1: **Hybrid ecDNA structure was validated by long DNA sequencing.**

Supplementary Fig. 2: **CRISPR interference targeting enhancers on hybrid ecDNA to SCC154 and NOKSI didn't block HPV oncogene expression.**

Supplementary Fig. 3: **JQ1 treatment on SCC154 did not significantly change E6/E7 expression.**

Supplementary Fig. 4: **Comparison of ChIP-seq, RNA-seq, and HiC-seq data between JQ1 treatment and DMSO in HMS001.**

Supplementary Fig. 5: **Short-time culture of PDX and multi-FISH between JQ1 treatment and DMSO in PDX_A, and comparison of HiC-seq data between JQ1 treatment and DMSO in PDX_A.**

Supplementary Tables:

Supplementary Table 1: **Copy number information of HPV in each sample.**

Supplementary Table 2: **gRNA information for CRISPRi.**

Supplementary Table 3: **Primer information for qPCR.**

S Fig. 1

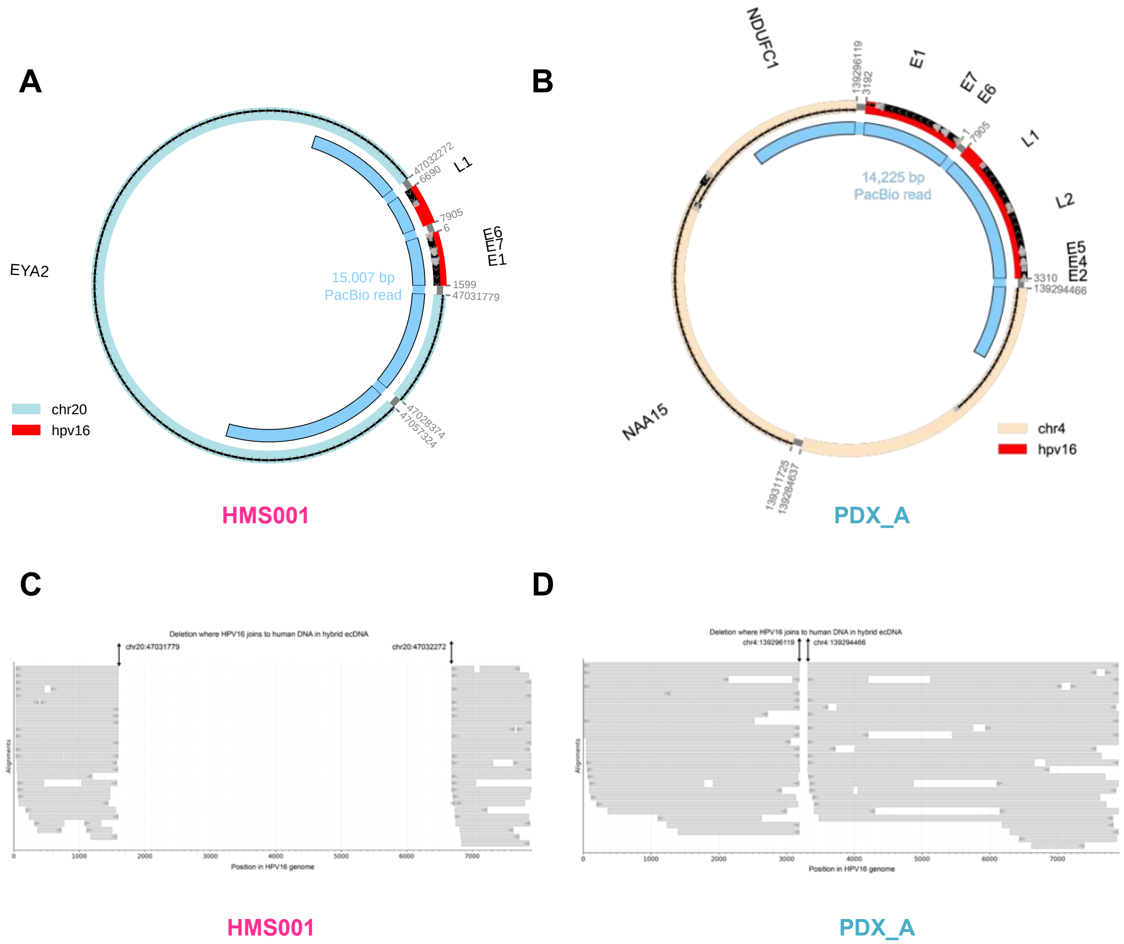

Supplementary Fig. 1. Hybrid ecDNA structure was validated by long DNA sequencing. Hybrid ecDNA structures of HMS001 and PDX_A were shown. 15,007 bp read and 14,225 bp read were aligned to hybrid sequence respectively (A and B). Pileup visualization of PacBio reads indicates location of deletions and where HPV16 joins to human DNA in hybrid ecDNA for HMS001 (C) and PDX_A (D).

S Fig. 2

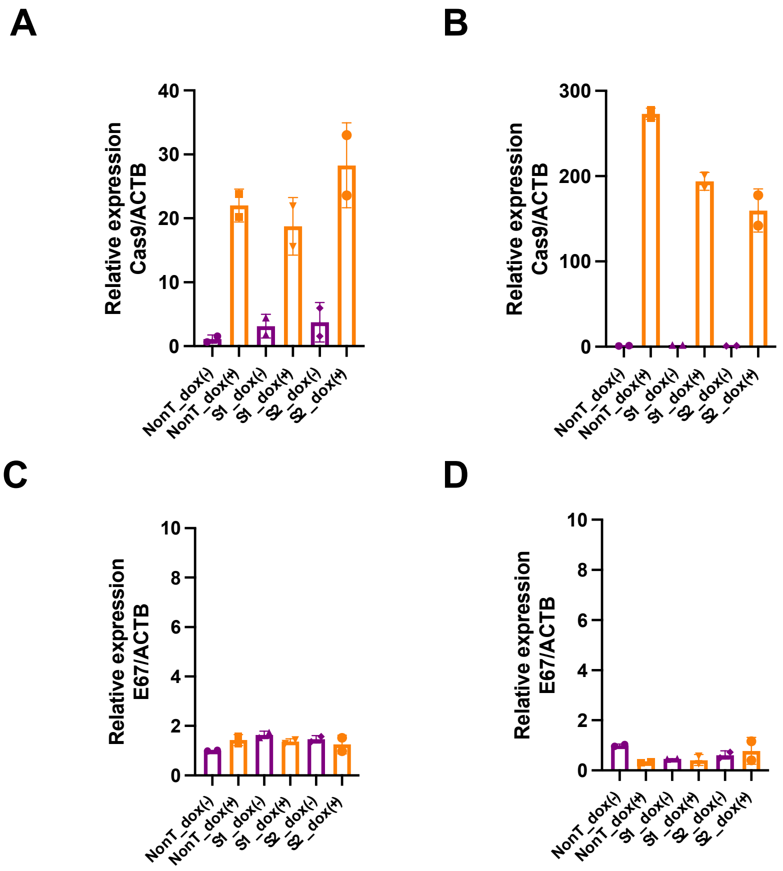

Supplementary Fig. 2. CRISPR interference targeting enhancers on hybrid ecDNA to SCC154 and NOKSI didn't block HPV oncogene expression. CRISPRi using dCas9-KRAB, targeting the enhancers (S1 and S2) on the hybrid ecDNA of HMS001 were performed for SCC154 and NOKSI. The expression of dCas9 in SCC154 and NOKSI after doxycycline induction was confirmed by qPCR (A and B). qPCR results of E6/E7 for SCC154 and NOKSI were shown. ACTB was used internal control. E6/E7 were not significantly changed in any CRISPRi condition (C and D, respectively). Two biological replicates were used and median with SD were shown. Source Data is available for panels A-D.

S Fig. 3

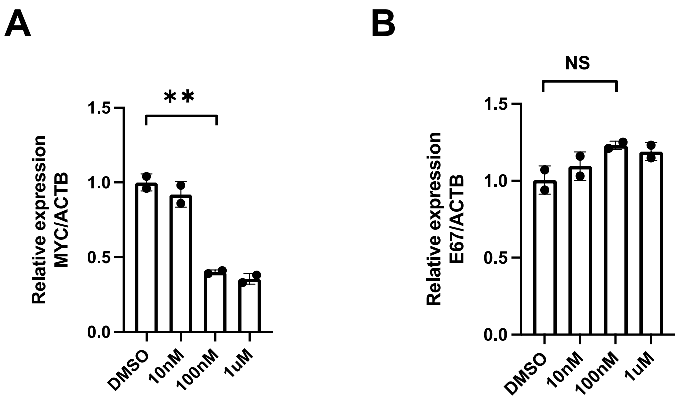

Supplementary Fig. 3. JQ1 treatment on SCC154 did not significantly change E6/E7 expression. qPCR results of MYC and E6/E7 were shown. ACTB was used internal control. MYC expressions were reduced after JQ1 treatment in a concentration-dependent manner, but E6/E7 were not changed after JQ1 treatment (A and B, ***P* = 0.005 and *P* = 0.08, respectively, two-tailed student's *t*-test). Two biological replicates were used and median with SD were shown. Source Data is available for panels A and B.

S Fig. 4

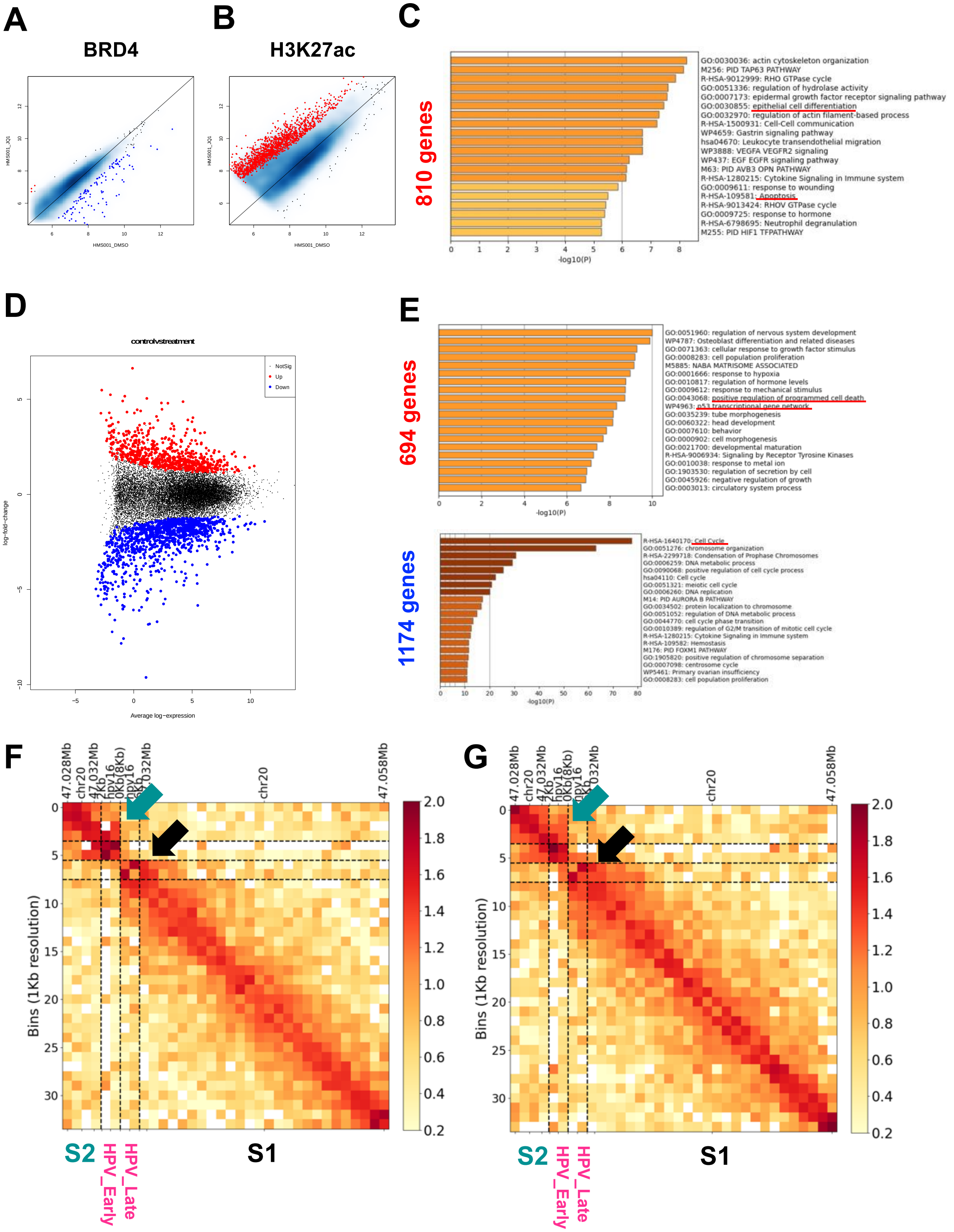
A
BRD4
HMS001_JQ1
HMS001_DMSO
B
H3K27ac
HMS001_JQ1
HMS001_DMSO
C
810 genes
GO:0030036: actin cytoskeleton organization
M256: PID TAP63 PATHWAY
R-HSA-9012999: RHO GTPase cycle
GO:0051336: regulation of hydrolase activity
GO:0007173: epidermal growth factor receptor signaling pathway
GO:0030855: epithelial cell differentiation
GO:0032970: regulation of actin filament-based process
R-HSA-1500931: Cell-Cell communication
WP4659: Gastrin signaling pathway
hsa04670: Leukocyte transendothelial migration
WP3888: VEGFA VEGFR2 signaling
WP437: EGF EGFR signaling pathway
M63: PID AVB3 OPN PATHWAY
R-HSA-1280215: Cytokine Signaling in Immune system
GO:0009611: response to wounding
R-HSA-109581: Apoptosis
R-HSA-9013424: RHOV GTPase cycle
GO:0009725: response to hormone
R-HSA-6798695: Neutrophil degranulation
M255: PID HIF1 TFPATHWAY
-log10(P)
D
controlvstreatment
NotSig
Up
Down
log-fold-change
Average log-expression
E
694 genes
GO:0051960: regulation of nervous system development
WP4787: Osteoblast differentiation and related diseases
GO:0071363: cellular response to growth factor stimulus
GO:0008283: cell population proliferation
M5885: NABA MATRISOME ASSOCIATED
GO:0001666: response to hypoxia
GO:0010817: regulation of hormone levels
GO:0009612: response to mechanical stimulus
GO:0043068: positive regulation of programmed cell death
WP4963: p53 transcriptional gene network
GO:0035239: tube morphogenesis
GO:0060322: head development
GO:0007610: behavior
GO:0000902: cell morphogenesis
GO:0021700: developmental maturation
R-HSA-9006934: Signaling by Receptor Tyrosine Kinases
GO:0010038: response to metal ion
GO:1903530: regulation of secretion by cell
GO:0045926: negative regulation of growth
GO:0003013: circulatory system process
-log10(P)
1174 genes
R-HSA-1640170: Cell Cycle
GO:0051276: chromosome organization
R-HSA-2299718: Condensation of Prophase Chromosomes
GO:0006259: DNA metabolic process
GO:0090068: positive regulation of cell cycle process
hsa04110: Cell cycle
GO:0051321: meiotic cell cycle
GO:0006260: DNA replication
M14: PID AURORA B PATHWAY
GO:0034502: protein localization to chromosome
GO:0051052: regulation of DNA metabolic process
GO:0044770: cell cycle phase transition
GO:0010389: regulation of G2/M transition of mitotic cell cycle
R-HSA-1280215: Cytokine Signaling in Immune system
R-HSA-109582: Hemostasis
M176: PID FOXM1 PATHWAY
GO:1905820: positive regulation of chromosome separation
GO:0007098: centrosome cycle
WP5461: Primary ovarian insufficiency
GO:0008283: cell population proliferation
-log10(P)
F
47.028Mb
chr20
47.032Mb
2Kb
hpv16
0Kb(8Kb)
hpv16
8Kb
47.032Mb
chr20
47.058Mb
Bins (1Kb resolution)
S2
HPV_Early
HPV_Late
S1
G
47.028Mb
chr20
47.032Mb
2Kb
hpv16
0Kb(8Kb)
hpv16
8Kb
47.032Mb
chr20
47.058Mb
Bins (1Kb resolution)
S2
HPV_Early
HPV_Late
S1

Supplementary Fig. 4. Comparison of ChIP-seq, RNA-seq, and HiC-seq data between JQ1 treatment and DMSO in HMS001. Differentially upregulated (red)/downregulated (blue) peaks of ChIP-seq for BRD4 or H3K27ac using HMS001 was shown (A and B, respectively). Gene Ontology analysis using 810 H3K27ac upregulated peaks with H3K4me1 peaks were shown (C). Differentially upregulated (red)/downregulated (blue) genes of RNA-seq using HMS001 with DMSO treatment or JQ1 treatment was shown (D). Gene Ontology analysis for differentially upregulated 694 genes and downregulated 1174 genes were shown (E). HiC-seq with DMSO treatment or JQ1 treatment was shown. HiC-seq showed no significant changes after JQ1 treatment in the interaction between S1 enhancer region and HPV late part (black allow) and between S2 and HPV early part (right green allow), suggesting that the structure of the hybrid ecDNA itself was not affected after JQ1 treatment (F and G)

S Fig. 5

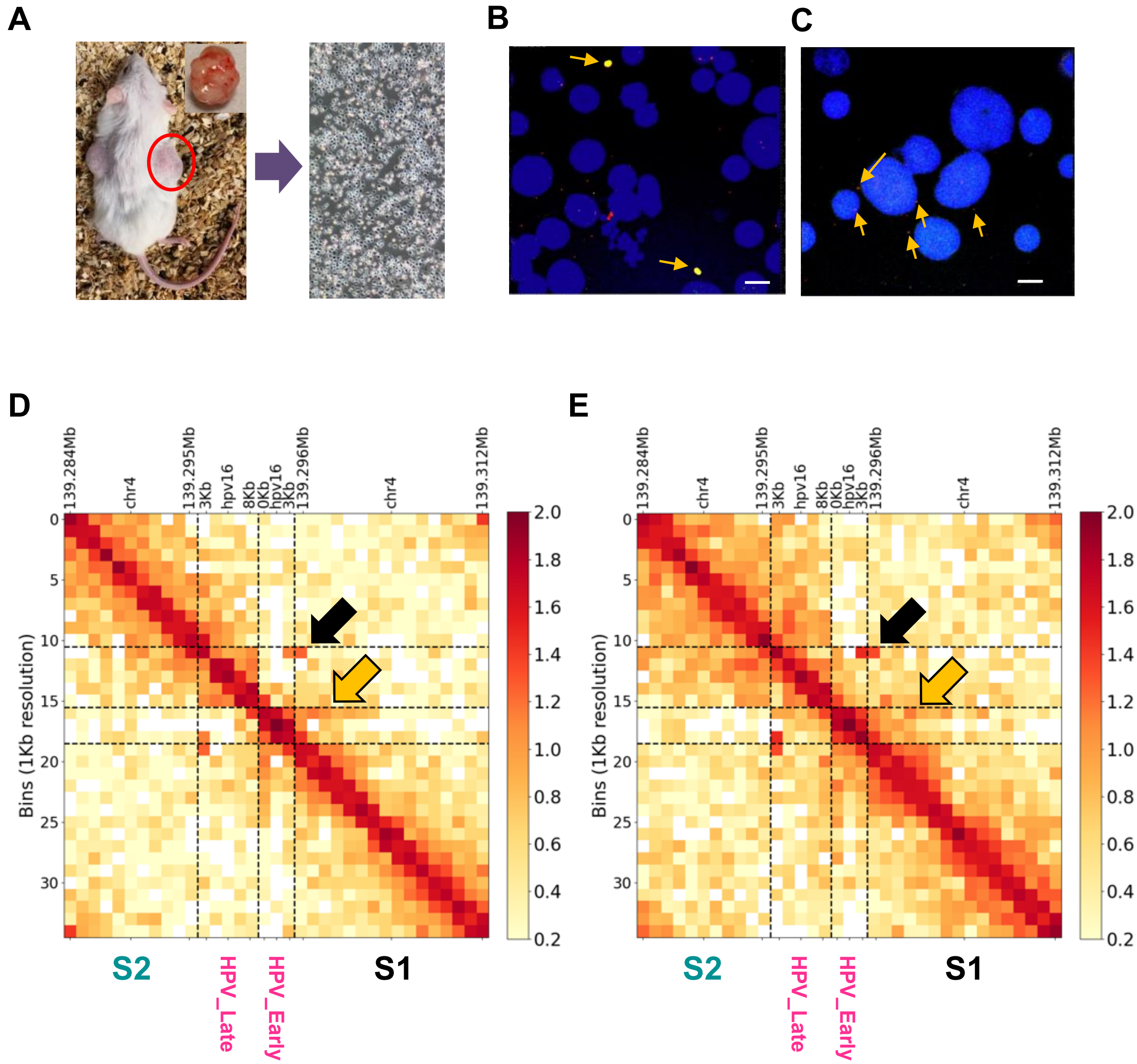

Supplementary Fig. 5. Short-time culture of PDX and multi-FISH between JQ1 treatment and DMSO in PDX_A, and comparison of HiC-seq data between JQ1 treatment and DMSO in PDX_A. Short-time culture of PDX_A tumor was created (A). Multi-probe FISH using NDUFC2 probe and HPV probe on hybrid ecDNA for short-time culture of PDX_A tumor is shown (B and C). Multi-FISH targeting hybrid ecDNA showed reduction of hybrid ecDNA FISH signals after JQ1 treatment. Each signal is green: NDUFC2, Red HPV, and blue: DAPI. Scale bar shows 10μm. HiC-seq with DMSO treatment or JQ1 treatment was shown. HiC-seq showed no significant changes after JQ1 treatment in the interaction between S1 enhancer region and HPV late part (black allow) and between S1 and HPV early part (yellow allow), suggesting that the structure of the hybrid ecDNA itself was not affected after JQ1 treatment (D and E).

S Table. 1

copynumber_hpv_samples_strain_cn

sample	HPV genome	HPV max copy number
**HMS001_hg38viral**	HPV16	4.13783705889601
**Noksi**	NONE IDENTIFIED	NA
**PDX_A_F3_DNA**	HPV16	4.10756382438681
**PDX_A_cell**	HPV16	4.688052399276280
**PDX_C_F3_DNA**	HPV16	41.3105258962521
**SCC154_combined**	HPV16	2.2921463017175100

Supplementary Table 1: **Copy number information of HPV in each sample.**

S Table. 2

gRNA_list		
gRNA_hybrid_ecDNA_enhancer_short_rank4		TAGTCAATACGACAACGAAT
gRNA_hybrid_ecDNA_enhancer_long_rank12		GTCTAAGTCAATCCATCCCG
nontargeting_gRNA		
NonTargetingControlGuideForHuman_0001	HGLibA_64384	ACGGAGGCTAAGCGTCGCAA

Supplementary Table 2: **gRNA information for CRISPRi.**

S Table. 3

ACTB_F	CACCATTGGCAATGAGCGGTTC
ACTB_R	AGGTCTTTGCGGATGTCCACGT
E67 F	CCGGTCGATGTATGTCTTGTT
E67 R	GAATGTCTACGTGTGTGCTTTG
dCas9_F	GCGAGCTGCAGAAAGGTAAC
dCas9_R	GCGAGGATCACTCTTTTGGA
cMYC Human qPCR F1	CCTGGTGCTCCATGAGGAGAC
cMYC Human qPCR R1	CAGACTCTGACCTTTTGCCAGG

Supplementary Table 3: **Primer information for qPCR.**
