# Supplementary material for: Inhibition of human-HPV hybrid ecDNA enhancers reduces oncogene expression and tumor growth in oropharyngeal cancer
Source: Nat Commun. 2025 Mar 26;16:2964. doi: 10.1038/s41467-025-57447-9 (PMC11947173; doi:10.1038/s41467-025-57447-9)
Supplement: Supplementary file 3 — Reporting Summary [file 41467_2025_57447_MOESM3_ESM.pdf]

nature portfolio

Corresponding author(s): Joseph Califano and Takuya Nakagawa

Last updated by author(s): 02/03/2025

Reporting Summary

Nature Portfolio wishes to improve the reproducibility of the work that we publish. This form provides structure for consistency and transparency in reporting. For further information on Nature Portfolio policies, see our Editorial Policies and the Editorial Policy Checklist.

Statistics

For all statistical analyses, confirm that the following items are present in the figure legend, table legend, main text, or Methods section.

n/a	Confirmed	
☐	☒	The exact sample size (n) for each experimental group/condition, given as a discrete number and unit of measurement
☐	☒	A statement on whether measurements were taken from distinct samples or whether the same sample was measured repeatedly
☐	☒	The statistical test(s) used AND whether they are one- or two-sided<br>*Only common tests should be described solely by name; describe more complex techniques in the Methods section.*
☒	☐	A description of all covariates tested
☐	☒	A description of any assumptions or corrections, such as tests of normality and adjustment for multiple comparisons
☐	☒	A full description of the statistical parameters including central tendency (e.g. means) or other basic estimates (e.g. regression coefficient) AND variation (e.g. standard deviation) or associated estimates of uncertainty (e.g. confidence intervals)
☐	☒	For null hypothesis testing, the test statistic (e.g. F, t, r) with confidence intervals, effect sizes, degrees of freedom and P value noted<br>*Give P values as exact values whenever suitable.*
☒	☐	For Bayesian analysis, information on the choice of priors and Markov chain Monte Carlo settings
☒	☐	For hierarchical and complex designs, identification of the appropriate level for tests and full reporting of outcomes
☐	☒	Estimates of effect sizes (e.g. Cohen's d, Pearson's r), indicating how they were calculated

Our web collection on statistics for biologists contains articles on many of the points above.

Software and code

Policy information about availability of computer code

Data collection	DNA was extracted using the QIAquick DNA mini kit (Qiagen, Hilden, Germany) for high-quality extraction per the manufacturer instructions.<br>RNA was extracted following the protocol of the Qiagen RNeasy Plus Mini Kit (Qiagen, Hilden, Germany).<br>High molecular weight DNA(>40kb) was extracted using the Nanobind CBB kit or Nanobing tissue kit (PacBio, Menlo Park, CA) following manufacturer's recommendations.<br>For ChIP experiments, 1x107 cells for cell lines, and 25mg tumors for PDX were used for each condition.<br>For ATAC-seq, around 50mg of pulverized PDX tumor tissue with liquid nitrogen and thawed cell lines (3x106) were used for nuclei preparation.<br>For HiC-seq, chromatin obtained from each cell line or PDX tumor was first cross-linked and digested using a restriction enzyme cocktail.

Data analysis	WGS data were analyzed using AmpliconSuite-pipeline, which encompasses AmpliconArchitect v1.3.r3 and AmpliconClassifier2 v0.5.1 to detect hybrid ecDNA, using the GRCh38_viral reference genome option (https://github.com/AmpliconSuite).<br>For ChIP-seq and ATAC-seq, Raw sequence data were first trimmed for sequencing adapters using fastp, then aligned to human genome (hg38) augmented by the HPV type 16 genome sequence as an extra chromosome (accession number AY686584.1), using STAR aligner35 with chimeric alignments allowed in the output. Peak calling was done in an unbiased way on an artificial sample created as the union of reads sampled randomly (with equal weight per sample) from all samples of the same type (ATAC-seq, H3K27ac, etc.), using HOMER (Hypergeometric Optimization of Motif EnRichment).<br>Hi-C data were processed by the runHiC python package (https://pypi.org/project/runHiC/) with a custom reference genome composed of human genome assembly hg38 augmented by the HPV type 16 genome as an extra chromosome. The runHiC package can remove duplicate reads, assign reads to restriction fragments, filter out invalid interaction pairs, and generate binned interaction matrices in *.mcool format. We specified the enzyme name as Arima in the filtering step to remove the read pair that maps to the same restriction fragment.

For manuscripts utilizing custom algorithms or software that are central to the research but not yet described in published literature, software must be made available to editors and reviewers. We strongly encourage code deposition in a community repository (e.g. GitHub). See the Nature Portfolio guidelines for submitting code & software for further information.

Data

Policy information about availability of data

All manuscripts must include a data availability statement. This statement should provide the following information, where applicable:

- Accession codes, unique identifiers, or web links for publicly available datasets
- A description of any restrictions on data availability
- For clinical datasets or third party data, please ensure that the statement adheres to our policy

Sequencing data of each cell line and PDX tumor data were submitted to the Sequence Read Archive (SRA) database under the accession number PRJNA1200897.
"AmpliconArchitect outputs associated with this study can be found at https://ampliconrepository.org/project/667314504677da1a8e41a4c0".
Code data for this study are available at:
https://github.com/virajbdeshpande/AmpliconArchitect
https://github.com/AmpliconSuite/CycleViz

Research involving human participants, their data, or biological material

Policy information about studies with human participants or human data. See also policy information about sex, gender (identity/presentation), and sexual orientation and race, ethnicity and racism.

Reporting on sex and gender	Sex was reported.
Reporting on race, ethnicity, or other socially relevant groupings	We did not report on race, ethnicity, or other socially relevant groupings.
Population characteristics	not applicable
Recruitment	HPVOPC patients who underwent surgery at Jacobs Medical Center at the University of California, San Diego (UCSD) Health were recruited.
Ethics oversight	Written informed consent was obtained from all patients. These samples were shared with the UCSD Human Research Protection Program (institutional review board (IRB)-approved protocol HRPP# 181755) by way of the Moores Cancer Center Biorepository and Tissue Technology resource.

Note that full information on the approval of the study protocol must also be provided in the manuscript.

Field-specific reporting

Please select the one below that is the best fit for your research. If you are not sure, read the appropriate sections before making your selection.

[x] Life sciences [ ] Behavioural & social sciences [ ] Ecological, evolutionary & environmental sciences

For a reference copy of the document with all sections, see nature.com/documents/nr-reporting-summary-flat.pdf

Life sciences study design

All studies must disclose on these points even when the disclosure is negative.

Sample size	The sample size for each experiment was determined based on pilot experiments and a review of the literature.
Data exclusions	No data exclusions were performed
Replication	All experiments were replicatd at least twice with consistent results.

Randomization	Mice were randomly assigned to control or treatment group.
Blinding	The investigators were not blinded to the allocation of each group during data collection and analysis.

Behavioural & social sciences study design

All studies must disclose on these points even when the disclosure is negative.

Study description	not applicable
Research sample	not applicable
Sampling strategy	not applicable
Data collection	not applicable
Timing	not applicable
Data exclusions	not applicable
Non-participation	not applicable
Randomization	not applicable

Ecological, evolutionary & environmental sciences study design

All studies must disclose on these points even when the disclosure is negative.

Study description	not applicable
Research sample	not applicable
Sampling strategy	not applicable
Data collection	not applicable
Timing and spatial scale	not applicable
Data exclusions	not applicable
Reproducibility	not applicable
Randomization	not applicable
Blinding	not applicable

Did the study involve field work? ☐ Yes ☒ No

Field work, collection and transport

Field conditions	not applicable
Location	not applicable
Access & import/export	not applicable
Disturbance	not applicable

Reporting for specific materials, systems and methods

We require information from authors about some types of materials, experimental systems and methods used in many studies. Here, indicate whether each material, system or method listed is relevant to your study. If you are not sure if a list item applies to your research, read the appropriate section before selecting a response.

Materials & experimental systems

n/a	Involved in the study
☐	☒ Antibodies
☐	☒ Eukaryotic cell lines
☒	☐ Palaeontology and archaeology
☐	☒ Animals and other organisms
☐	☒ Clinical data
☒	☐ Dual use research of concern
☒	☐ Plants

Methods

n/a	Involved in the study
☐	☒ ChIP-seq
☒	☐ Flow cytometry
☒	☐ MRI-based neuroimaging

Antibodies

Antibodies used

H3K4me3 (#9751, 2µg, Cell Signaling Technology, Danvers, MA)
H3K4me1 (#C5326S, 2µg, Cell Signaling Technology)
H3K27ac (#91194, 4µg, Active Motif, Carlsbad, CA)
IgG (#2729, 2µg, Cell Signaling Technology)
BRD4 (#A301-985A50, 7.5µg, Bethyl Laboratories, Montgomery, TX)
Cas9 (#14697S, 1:1000, Cell Signaling Technology)
MYC (#13987S, 1:1000, Cell Signaling Technology)
E6 (#GTX132686, 1:100, GeneTex, Irvine, CA)
E7 (#GTX133411, 1:100, GeneTex)
GAPDH (#2118S, 1:6000, Cell Signaling Technology)

Validation

All antibodies we used in this study are commercially available. Manufacturer's suggested experimental parameters were used.

Eukaryotic cell lines

Policy information about cell lines and Sex and Gender in Research

Cell line source(s)

SCC154 was purchased from the American Type Culture Collection (ATCC: CRL-3241). HMS001 was a gift from the James Rocco lab (Ohio State). NOKSI was a gift from the lab of Silvio Gutkind.

Authentication

Authentication by short tandem repeat (STR) profile of each cell line was performed by the original developer.

Mycoplasma contamination

Mycoplasma testing was conducted monthly using the MycoAlert-Plus Mycoplasma Detection Kit (Lonza) and all cell lines were confirmed negative.

Commonly misidentified lines
(See ICLAC register)

not applicable

Palaeontology and Archaeology

Specimen provenance

not applicable

Specimen deposition

not applicable

Dating methods

not applicable

☐ Tick this box to confirm that the raw and calibrated dates are available in the paper or in Supplementary Information.

Ethics oversight

Palaeontology and Archaeology

Note that full information on the approval of the study protocol must also be provided in the manuscript.

Animals and other research organisms

Policy information about studies involving animals; ARRIVE guidelines recommended for reporting animal research, and Sex and Gender in Research

Laboratory animals	Female 4-week-old nonobese diabetic/severe combined immunodeficiency (NOD/SCID) mice were purchased from The Jackson Laboratory.
Wild animals	not applicable
Reporting on sex	Female
Field-collected samples	not applicable
Ethics oversight	The animal study was approved by the Institutional Animal Care and Use Committee (IACUC) of the University of California, San Diego (UCSD). The protocol number is S16200 with Dr Joseph Califano as principal investigator.

Note that full information on the approval of the study protocol must also be provided in the manuscript.

Clinical data

Policy information about clinical studies

All manuscripts should comply with the ICMJEguidelines for publication of clinical research and a completedCONSORT checklist must be included with all submissions.

Clinical trial registration	not applicable
Study protocol	not applicable
Data collection	We used PDX_A: 55-year-old male, PDX_C: 76-year-old male, and PDX004: 75-year-old male.
Outcomes	not applicable

Dual use research of concern

Policy information about dual use research of concern

Hazards

Could the accidental, deliberate or reckless misuse of agents or technologies generated in the work, or the application of information presented in the manuscript, pose a threat to:

No	Yes	
☒	☐	Public health
☒	☐	National security
☒	☐	Crops and/or livestock
☒	☐	Ecosystems
☒	☐	Any other significant area

Experiments of concern

Does the work involve any of these experiments of concern:

No	Yes	
☒	☐	Demonstrate how to render a vaccine ineffective
☒	☐	Confer resistance to therapeutically useful antibiotics or antiviral agents
☒	☐	Enhance the virulence of a pathogen or render a nonpathogen virulent
☒	☐	Increase transmissibility of a pathogen
☒	☐	Alter the host range of a pathogen
☒	☐	Enable evasion of diagnostic/detection modalities
☒	☐	Enable the weaponization of a biological agent or toxin
☒	☐	Any other potentially harmful combination of experiments and agents

Plants

Seed stocks	not applicable
Novel plant genotypes	not applicable
Authentication	not applicable

ChIP-seq

Data deposition

- [x] Confirm that both raw and final processed data have been deposited in a public database such as GEO.
- [x] Confirm that you have deposited or provided access to graph files (e.g. BED files) for the called peaks.

Data access links<br>*May remain private before publication.*	Sequencing data of each cell line and PDX tumor data were submitted to the Sequence Read Archive (SRA) database under the accession number PRJNA1186352.
Files in database submission	bedGraph or bigwig files and fastq files
Genome browser session<br>(e.g. UCSC)	not applicable

Methodology

Replicates	For the H3K27ac mark, we have two replicates each of HMS001 and PDX_A treated with either vehicle or JQ1
Sequencing depth	30M-45M/sample
Antibodies	Antibody for each immunoprecipitation using H3K4me3 (#9751, 2µg, Cell Signaling Technology, Danvers, MA), H3K4me1 (#C5326S, 2µg, Cell Signaling Technology), H3K27ac (#91194, 4µg, Active Motif, Carlsbad, CA), BRD4 (#A301-985A50, 7.5µg, Bethyl Laboratories, Montgomery, TX), and IgG for negative control was used.
Peak calling parameters	Peak calling was done in an unbiased way on an artificial sample created as the union of reads sampled randomly (with equal weight per sample) from all samples of the same type (K4me1, H3K27ac, etc.), using HOMER (Hypergeometric Optimization of Motif EnRichment).
Data quality	Peak quantitation was done for each sample individually by counting reads aligning to the peak regions determined in the previous step.
Software	HOMER (Hypergeometric Optimization of Motif EnRichment)

Flow Cytometry

Plots

Confirm that:

- [ ] The axis labels state the marker and fluorochrome used (e.g. CD4-FITC).
- [ ] The axis scales are clearly visible. Include numbers along axes only for bottom left plot of group (a 'group' is an analysis of identical markers).
- [ ] All plots are contour plots with outliers or pseudocolor plots.
- [ ] A numerical value for number of cells or percentage (with statistics) is provided.

Methodology

Sample preparation	*Describe the sample preparation, detailing the biological source of the cells and any tissue processing steps used.*
Instrument	*Identify the instrument used for data collection, specifying make and model number.*
Software	*Describe the software used to collect and analyze the flow cytometry data. For custom code that has been deposited into a community repository, provide accession details.*

Cell population abundance | *Describe the abundance of the relevant cell populations within post-sort fractions, providing details on the purity of the samples and how it was determined.*

Gating strategy | *Describe the gating strategy used for all relevant experiments, specifying the preliminary FSC/SSC gates of the starting cell population, indicating where boundaries between "positive" and "negative" staining cell populations are defined.*

☐ Tick this box to confirm that a figure exemplifying the gating strategy is provided in the Supplementary Information.

Magnetic resonance imaging

Experimental design

Design type | *Indicate task or resting state; event-related or block design.*

Design specifications | *Specify the number of blocks, trials or experimental units per session and/or subject, and specify the length of each trial or block (if trials are blocked) and interval between trials.*

Behavioral performance measures | *State number and/or type of variables recorded (e.g. correct button press, response time) and what statistics were used to establish that the subjects were performing the task as expected (e.g. mean, range, and/or standard deviation across subjects).*

Acquisition

Imaging type(s) | *Specify: functional, structural, diffusion, perfusion.*

Field strength | *Specify in Tesla*

Sequence & imaging parameters | *Specify the pulse sequence type (gradient echo, spin echo, etc.), imaging type (EPI, spiral, etc.), field of view, matrix size, slice thickness, orientation and TE/TR/flip angle.*

Area of acquisition | *State whether a whole brain scan was used OR define the area of acquisition, describing how the region was determined.*

Diffusion MRI ☐ Used ☐ Not used

Preprocessing

Preprocessing software | *Provide detail on software version and revision number and on specific parameters (model/functions, brain extraction, segmentation, smoothing kernel size, etc.).*

Normalization | *If data were normalized/standardized, describe the approach(es): specify linear or non-linear and define image types used for transformation OR indicate that data were not normalized and explain rationale for lack of normalization.*

Normalization template | *Describe the template used for normalization/transformation, specifying subject space or group standardized space (e.g. original Talairach, MNI305, ICBM152) OR indicate that the data were not normalized.*

Noise and artifact removal | *Describe your procedure(s) for artifact and structured noise removal, specifying motion parameters, tissue signals and physiological signals (heart rate, respiration).*

Volume censoring | *Define your software and/or method and criteria for volume censoring, and state the extent of such censoring.*

Statistical modeling & inference

Model type and settings | *Specify type (mass univariate, multivariate, RSA, predictive, etc.) and describe essential details of the model at the first and second levels (e.g. fixed, random or mixed effects; drift or auto-correlation).*

Effect(s) tested | *Define precise effect in terms of the task or stimulus conditions instead of psychological concepts and indicate whether ANOVA or factorial designs were used.*

Specify type of analysis: ☐ Whole brain ☐ ROI-based ☐ Both

Statistic type for inference | *Specify voxel-wise or cluster-wise and report all relevant parameters for cluster-wise methods.*

(See Eklund et al. 2016)

Correction | *Describe the type of correction and how it is obtained for multiple comparisons (e.g. FWE, FDR, permutation or Monte Carlo).*

Models & analysis

n/a	Involved in the study
☒	☐ Functional and/or effective connectivity
☒	☐ Graph analysis
☒	☐ Multivariate modeling or predictive analysis

Functional and/or effective connectivity	*Report the measures of dependence used and the model details (e.g. Pearson correlation, partial correlation, mutual information).*
Graph analysis	*Report the dependent variable and connectivity measure, specifying weighted graph or binarized graph, subject- or group-level, and the global and/or node summaries used (e.g. clustering coefficient, efficiency, etc.).*
Multivariate modeling and predictive analysis	*Specify independent variables, features extraction and dimension reduction, model, training and evaluation metrics.*
